# Implementation and Evaluation of a Patient-Reported Health Status Survey for Survivors of Childhood Malignancies Treated with Radiation †

**DOI:** 10.3390/cancers17223634

**Published:** 2025-11-12

**Authors:** Jaitri Joshi, Miranda Lawell, Keith Allison, Benjamin Bajaj, Sara Dennehy, Melanie Rose, Nancy Tarbell, Torunn Yock

**Affiliations:** Department of Radiation Oncology, Massachusetts General Hospital, Harvard Medical School, Boston, MA 02114, USAmlawell@mgh.harvard.edu (M.L.); kwallison@mgb.org (K.A.); bbajaj@mgb.org (B.B.); sdennehy2@mgh.harvard.edu (S.D.); mlrose@mgb.org (M.R.); ntarbell@mgh.harvard.edu (N.T.)

**Keywords:** pediatric oncology, radiotherapy, patient-reported outcomes, survivorship

## Abstract

**Background/Objectives:** Long-term follow up of pediatric radiotherapy survivors referred to tertiary and quaternary centers is difficult, limiting outcome monitoring. We implemented an electronic Health Status Survey (HSS) to capture survivorship data. **Methods:** The Health Status Survey (HSS) was distributed to survivors aged 1 month–25 years treated with curative-intent radiotherapy at our institution. Eligibility required confirmed survival and valid email contact. Survey responses and demographics were summarized with descriptive statistics and comparative tests. Surveys were sent to 876 eligible participants, with 322 responses received (36.8%) between December 2023 and March 2024. **Results:** Survey completion extended follow up by a median of 18.3 months (about 1.5 years), improving monitoring beyond the last chart-documented visit. Respondents reported 12 recurrences, 5 s malignancies, and 7 deaths not captured in prior records, altering event-free survival statistics by as much as 7.5%. Most respondents (90.1%) reported a medical visit in the past year, and many provided updates on ongoing health issues, provider contact, and social outcomes. **Conclusions:** The HSS is a replicable method for extended follow up of tertiary and quaternary care center patients, capturing previously unreported clinical, social, and educational outcomes. By supplementing chart data with patient-reported information, this cost-effective tool supports targeted annual follow up and the identification of trends for long-term survivorship care.

## Simple Summary

For pediatric patients, the survival and health events after radiation treatment for cancer are especially important to track long-term. However, patients receiving radiation often do so at centers outside of the institution they regularly visit for healthcare. This makes progress-tracking difficult after treatment is complete. To combat this, we created a replicable web-based Health Status Survey to reconnect with patients that had previously received radiation for pediatric cancers at our institution. The survey gathered updates about symptoms and health updates as well as educational and social well-being. Sending out this survey increased our knowledge of health outcomes for our patients by over 18 months in comparison to latest updates on medical chart review. The outcomes found from this survey included twenty-four major health events like cancer recurrence and new, related, cancer diagnoses. This research demonstrates that the HSS is a cost-efficient tool for long-term monitoring, ensuring survival data is accurate, and helping specialized centers better understand the evolving needs of survivors.

## 1. Introduction

Pediatric malignancies remain the second largest cause of childhood fatalities despite a recent increase in survivorship [1]. The treatment of pediatric malignancies has notably changed with innovations in radiation therapy. Radiation interventions for pediatric malignancies are often used as adjuvant therapies with initial surgical resection and chemotherapy. The extent of radiation use in a multimodal treatment approach to pediatric malignancies is determined by factors such as cancer subtype and staging. The heterogeneous nature of the malignancies within the pediatric cohort of patients, combined with the relatively limited large-scale survivorship data for those treated with recent innovations in therapeutics, has led to difficulty in monitoring the needs of patients after treatment. Radiation therapy is offered across tertiary and quaternary care centers, with treatment modality selection often determining the appropriate care setting. Tertiary care centers are sub-specialty medical sites to which quaternary care sites provide extension for even greater specialization. Patients are predominately referred from their home institutions to radiation centers to receive care for a relatively short period of time. After radiation treatment, patients mainly continue to follow up and receive additional treatments as needed for their diagnosis at their referring institution. As a result, consistent follow up with the radiation treatment center becomes difficult, limiting survivorship and health outcome data available to understand treatment efficacy and long-term effects. Survivors of childhood cancer have a higher burden of critical care admissions and in-patient services in the decades following treatment in comparison to matched community controls [2,3]. Younger patients, as a result of their cellular sensitivity to radiation and stage of development, may face higher risks of developing adverse effects/sequelae [3,4]. Considerations for optimization of long-term quality-of-life in younger patients have therefore been evaluated with the alternative of avoiding or delaying radiation therapy against medical management. It is in this context of healthcare utilization patterns and support needed by radiation-treated survivors, that a short-form data collection methodology becomes pertinent to employ. With the shift into adulthood for pediatric patients, maintaining contact with the patient population to understand the specific needs and barriers faced by this group is best accomplished by a modified QoL assessment survey. The needs of survivors may differ between those who are still minors today and those who have transitioned to adulthood. Designing a survey that is self-administered by patients annually allows institutional understanding of specific patient-case-related updates as well as an analysis of trends within the growing population of survivors of pediatric malignancies. This paper seeks to describe the implementation of a self-administered assessment, the Health Status Survey, for gathering longitudinal clinical data related to QoL measures for radiation therapy-treated survivors of pediatric malignancies. In addition, this paper aims to report on the insights provided by the survey that are most pertinent to development of clinical support and consistent follow up for childhood survivors treated with radiation therapy into adulthood.

## 2. Materials and Methods

### 2.1. Patient Inclusion and Survey Eligibility

The Health Status Survey (HSS) was designed and submitted to the Massachusetts General Brigham Institutional Review Board (IRB) for approval. The survey design and REDCap database used are as described in the pilot study [5], Rose and Lawell et al. (2025). The current iteration of the survey was created for email distribution and solely web-based completion. Previously, the pilot distribution of the HSS included a combination of email, phone call, and physical mail. Patients who were eligible to receive an HSS included English-speakers between the ages of 1 month and 25 years at the time of treatment, who received radiotherapy with a curative intent at Massachusetts General Hospital, who were not reported to be deceased or to have an active recurrent status at time the survey was sent, and who had an email address on record. Select patients with recurrence/second malignancies that were well-controlled with treatment were included at provider discretion. Patients were invited to complete the survey between December 2023 and March 2024. The survey invitation email greeted the patient and their family and expressed interest in receiving an update about the patient. Links for the survey were generated by REDCap and were individualized to each patient. The survey is provided in its entirety in Appendix A. The survey link in the invitation email directed the participant to the survey on the REDCap platform.

### 2.2. Overview of the Health Status Survey

The survey consists of 11 questions at minimum and 30 questions at maximum. The true number of questions presented to each respondent was dependent on the level of survey expansion from the branching logic feature. Before initiating distribution, patient information in the REDCap database including disease status, last date of follow up, and contact information were updated using patient medical records in EPIC and within Epic’s Care Everywhere function for external site records. An additional internet search was conducted for patient obituaries to minimize risk of sending surveys to patients who were deceased and may not yet have a status update in EPIC or the patient database on REDCap. With the updated REDCap database information, patients who had no follow up after treatment completion at Massachusetts General Hospital, significant medical circumstances surrounding tumor recurrence, and/or significant psychosocial difficulties were excluded at the study team and medical provider’s discretion. Eligible patients and families were sent the Health Status Survey invitations electronically within the encrypted REDCap system through a Massachusetts General Hospital platform electronic mailbox created for this study. All surveys were sent at 6:00 p.m. EST. All electronic communication followed protocol for protecting participant PHI. An automatic email reminder was generated to be sent by REDCap after six days if no survey response was received. A maximum of three reminders were sent to each patient. The electronic mailbox was monitored daily. In case of a response indicating the patient’s death or one indicating that the patient would no longer be like to be contacted, patients were removed from further survey reminders and the MGH electronic medical record was updated to reflect this.

### 2.3. Statistical Analysis

The SPSS Statistics software suite by IBM version 29.0.1.1 was used to conduct all statistical analyses. Descriptive statistics including counts and percentages (N, %) of categorical data and medians (min–max) of numerical data were used to summarize survey responses and existing patient demographics. A chi-square test for independence was conducted to assess the likelihood of changes to contact information between age status groups; symptoms and specialty visits reported by treatment site; specialty visits by age at radiation. A Fischer’s Exact Test was used for counts under 5. All *p*-values are based on a two-sided hypothesis with a significance level of 0.05.

## 3. Results

### 3.1. Description of the Eligible Population and Responding Study Population

Of the 928 distributed surveys to eligible patients and families from December 2023 to March 2024, 52 were undeliverable to the email on the database. Therefore, 876 patients were sent emails inviting them to complete the Health Status Survey. From the 876 eligible patients and families who were sent a survey email, 322 responses were received, a response rate of 36.8%. The 322 patients who responded formed the study population. Table 1 displays the characteristics of the respondents, with Table 1a including demographics and Table 1b focusing on tumor subtype breakdown for CNS and non-CNS radiation-treated cohorts. Table 1b includes the proportion of CNS-tumor patients who received CSI (craniospinal irradiation), in which patients with Medulloblastoma/PNET/Pineoblastoma, Ependymoma, Germ Cell, Glial Tumors/Astrocytoma, Craniopharyngioma, and Vascular Lesion subtypes were included.

### 3.2. Contact Information Updates Varied by Age

Of the 322 total respondents, 185 (57.8%) reported a change in any of their preferred contact information (residential address, phone number, or email) from the entries captured in the database. The proportion of patients reporting a change in their preferred contact information differed significantly by age group: 47.6% for the Minor at RT, Minor at Survey group; 65.0% for the Minor at RT, Adult at Survey group; and 71.0% for the Adult at RT, Adult at Survey group (*p* = 0.002). Results are displayed in Table 2.

### 3.3. Reminders and Extension of Follow Up

**Figure 1:**
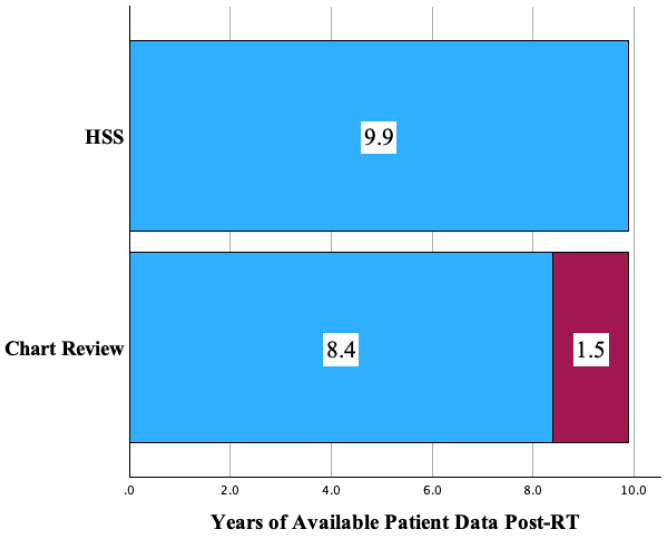
Extension of follow up (years) with the addition of HSS surveillance to primary chart review.

A majority (75.9%) of the overall respondent population was captured prior to receiving a second reminder (17.7%) or third (6.3%) reminder. The extension of follow up represents the time until the most current data the HSS provided beyond what was available in the local medical record of patients and was a median of 18.3 months (1.5 years) for respondents. Despite the response rate of only 36.8%, follow up on this cohort was extended from 8.4 years to 9.9 years (range of 0 to 20.0 years) (Figure 1).

### 3.4. Medical Visits and Issues in the Past Year

**Figure 2:**
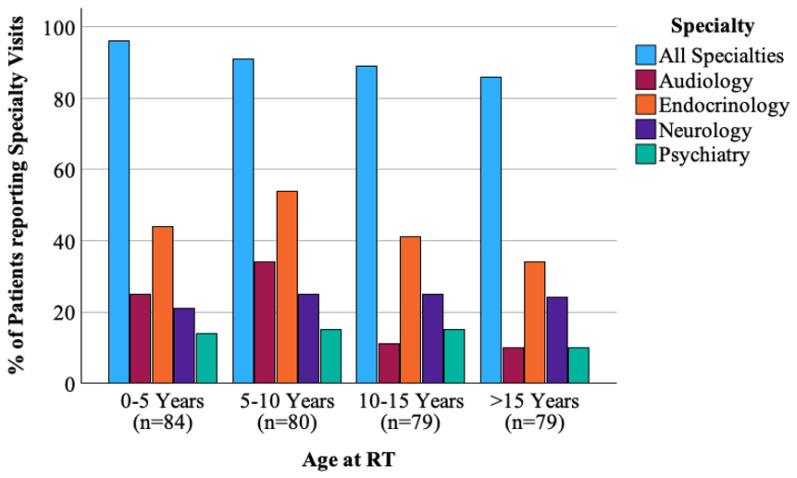
Proportion of patients reporting specialty visits in the past year by age at time of radiation.

**Figure 3:**
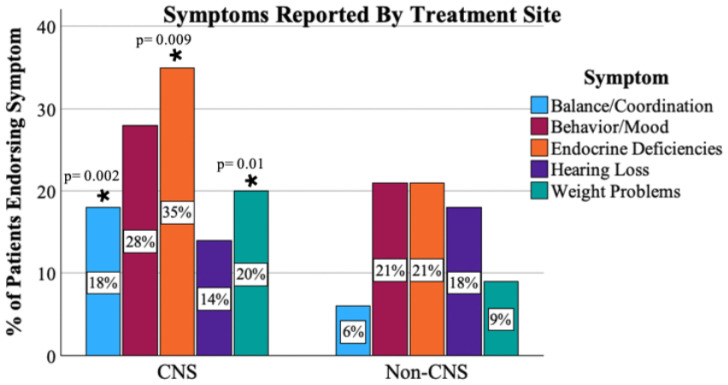
Proportion of patients endorsing symptoms by primary tumor type. *: difference (between CNS and non-CNS) of statistical significance.

Two-hundred ninety (90.1%) respondents endorsed visiting a medical provider in the past year. However, only 45% of these patients had a medical note accessible to the research team within the past 12 months upon chart review. The types of medical specialists the patients visited overall were endocrinologists (47%), ophthalmologists (36%), neurologists (27%), hearing specialists/audiologists (22%), and psychologists/psychiatrists (16%) (Figure 2). The majority, 227 (71.8%), reported that they had been experiencing medical problems with the most common being endocrine deficiencies [e.g., low thyroid, growth, or sex hormone] (*n* = 99, 31%), behavior/mood-related problems [e.g., anxiety, depression] (*n* = 83, 26%), and fatigue (*n* = 78, 24%). Patients with CNS tumors were significantly more likely to report balance (*p* = 0.002), endocrine (*p* = 0.009), and weight problems (*p* = 0.01) than patients with tumors outside of the CNS. There were no differences by CNS vs. non-CNS tumor types in patients for medical visits for behavior/mood issues (*p* = 0.24) or for hearing loss (*p* = 0.47). Figure 3 displays the response profile of medical problems stratified by treatment site.

**Figure 4:**
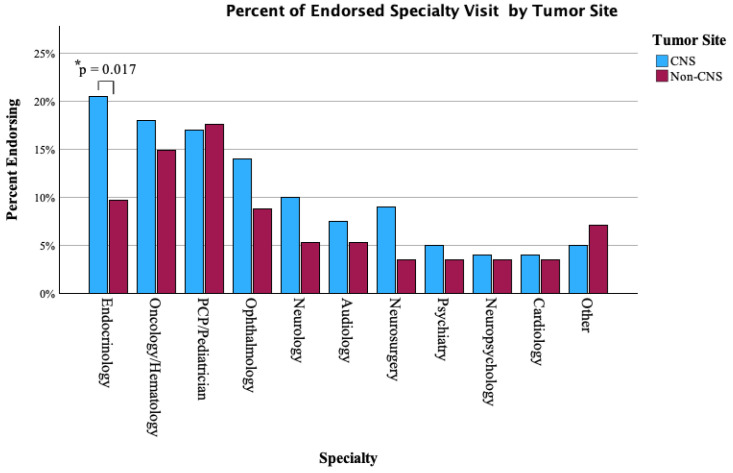
Proportion of CNS vs. non-CNS patients endorsing sub-specialty visits in the past year. *: difference (between CNS and non-CNS) of statistical significance.

Specialty visit patterns varied between CNS-treated and non-CNS patients. Endocrinology (20.5% vs. 9.7%, *p* = 0.017) and visits were significantly more frequent among CNS-treated patients. Rates of ophthalmology visits (14.0% vs. 8.8%), audiology (7.5% vs. 5.3%), psychiatry (5.0% vs. 3.5%), and other specialties were similar and not significant between groups. Full specialty visit comparisons are shown in Figure 4.

### 3.5. Prevalence of Recurrence, Second Malignancies, and Deaths Discovered

During the electronic medical record review prior to survey distribution, three recurrences and four second tumors were captured. After survey distribution, an additional twelve patients reported recurrences, five additional patients reported second tumors, and seven patients were reported deceased by their families. In summary, the HSS uncovered 24 (7.5%) major health/disease events previously not known.

### 3.6. Social and Educational Status of Patients Post Treatment

A total of 224 patients were enrolled in school at the time of survey completion, of which 134 (60%) reported receiving additional school support. The two most common categories were classroom accommodations (50%) and curriculum modification (16%). The other reported categories were social/emotional support (13%); speech/physical/occupational therapy (12% each); adaptive physical education (8%); 1:1 aid (5%); and 8% miscellaneous. Of the 87 respondents above age 18 who reported they were not enrolled in school, 17 (19.5%) were not employed, 14 (16.15%) were employed part-time, and 56 (64.4%) reported full-time employment. Of the 70 patients who reported full-time or part-time employment, 46 provided details. The main industries reported included skilled trades with 6 (13%), science/technology/engineering/mathematics (4, 8.7%), hospitality (15, 32.6%), education/work with children (10, 21.7%), health/social services (5, 10.9%), and business (6, 13%).

The final question of the survey prompted patients to “please share an area of interest, exciting adventure, or accomplishment from the past year”. Responses to this free-text prompt were provided by 254 (78%) patients. Many patients shared educational achievements such as college admissions or graduation, while others shared milestones of engagement, marriage, or having children.

## 4. Discussion

The Health Status Survey was designed to collect health, social, and educational outcome measures for survivors of pediatric malignancies. With the rising number of survivors treated at tertiary/quaternary centers for radiation, the challenge of follow up has impeded upon the collection of long-term health outcome information. This study demonstrated improved follow up time for respondents by an average of 1.5 years and revealed critical information that could alter event-free survival (EFS) statistics by as much as 7.5%. EFS is commonly reported in pediatric and adult cancer studies and comprises recurrences, second tumors, and deaths. These events were not otherwise known—despite medical record review at the quaternary institution that treated these patients. Quaternary care facilities, like proton centers, have a high rate of externally referred patients and would benefit most from electronic methods of health status data collection [5]. The HSS leverages patient-reported outcomes and provides a cost-effective method to update information regarding patients’ provider contact and institution as well as critical information on recent medical and social health metrics.

### 4.1. Responses Received and Limitations of Response Rate

The response rate of patients to the HSS provides an indication of the level of engagement with web-based survey interventions for childhood cancer survivors. The response rate (36.8%) is close to the average rates seen for recipients of web-based patient-reported outcomes surveys [6,7]. A review of response rates in registry-based studies noted that an electronic-only survey format had a response rate of 42% ± 8.7. In the initial formation of the study group, 928 emails were sent to patients on the institutional database, of which 52 were known to be undeliverable from the autoreply function—but it is unclear how many were no longer used. This cohort of patients who were out of contact using the survey sending method emphasizes the need for consistent follow up to update contact information. Though representing only 5% of the total eligible cohort, these undeliverable emails represent outdated or incorrect database information and may have the potential to compromise the representativeness of the survivor population involved in the HSS. Continued annual HSS distribution can mitigate this effect with email, phone number, and address validation.

Though the in-person health survey distribution may have potential for higher response rates, the constraints associated with the widespread geographic range and low rates of treating-institution affiliation post treatment mitigate the potential advantages of this method [8,9,10]. E-mail distribution, rather than phone calls which were also a collected type of contact information for patients, has benefits of standardization of responses, lower risk of errors in transferring collected information to a database, and ability to complete the survey at the patient or guardian’s availability. The e-mail delivery also allows for consistent reminders to be sent to patients with minimized burden on the clinical study staff. The median response time of 6.1 days indicated the utility of automated reminders as a reminder was sent after exactly 6.0 days from the initial survey invitation. Furthermore, most respondents preferred e-mail distribution, although importantly, the second most preferred contact method was SMS/text survey invitations. Those who demonstrated this preference were asked to sign for consent to receive messages related to future HSS distribution. Survey distribution method may be less of a limiting factor to response rate in future HSS distribution using both updated email and SMS modalities.

### 4.2. Follow Up Extended by the HSS

One of the most critical measures for utility of HSS implementation is the extension of follow up time. The extension of follow up time is a measure of the period that had passed from the latest follow up in chart review to the survey response date. Chart review completed prior to distribution of the HSS set a baseline follow up of 8.4 years. The HSS prolonged monitoring of health updates for a median of 18.3 months, about 1.5 years, to 9.9 years. This extension of health updates for the study respondents allows important information to be collected about late side effects of treatment as well as disease status.

### 4.3. Recurrence, Second Malignancies, and Deaths

Recurrences, second tumors/malignancies, and deaths are critical events in oncology that inform investigators of the efficacy of the treatment. The HSS shows high value as a supplement to conventional surveillance with manual chart review with faster reports of changes to disease-free survival, especially with yearly HSS distribution. Many patients also included the name of their provider and institution at which they are followed, which can further enable accurate follow up.

Surveys were not sent to deceased patients or their guardians, and this status was searched for using chart reviews and internet searches for obituaries. Despite these efforts, the seven parent responses included seven deaths of which we were not aware. While this is not an optimal way to identify patient deaths, it is an important health outcome critical to cancer research and helps demonstrate the value of patient- or parent-reported outcomes.

### 4.4. Health Management Practices

The health issues reported among irradiated survivors varied according to where the tumor arose, in the CNS or outside of the CNS. Patients with CNS tumors reported greater difficulties with endocrine deficiencies, balance difficulties, and weight problems, which is consistent with the literature [11,12,13,14,15,16,17,18,19,20]. Craniospinal irradiation for CNS malignancies may cause direct, dose-dependent, radiation injury to the hypothalamic–pituitary axis and adjacent structures including the cerebellum, brainstem, and thyroid gland due to the dose localization of the radiation therapy. The hypothalamic–pituitary region, as an important regulator of gonadotropins and the growth, adrenocorticotropic, and thyroid hormones, contributes to abnormalities in these endocrine function indicators when disrupted. Accordingly, in our study, CNS-treated patients reported significantly higher endocrinology visits in the past year in comparison to non-CNS-treated patients. Though dose received was not a point of focus for the current analysis, future applications of the survey may be used in conjunction with available patient data (such as cumulative dose received to relevant structures) to further investigate the differences to self-reported endocrine sequelae [4,18,19]. Additional context including chemotherapeutic dosing may also help explain differences in endocrine sequelae between patients. Endocrine disruptions are seen with chemotherapeutics for oncological tumor control including gonadal failure with alkylating agent use and thyroid dysfunction with tyrosine kinase inhibitors [19]. Hypothalamic control of satiety and fatigue-related pathways, in addition, complicates concerns of weight in conjunction with the growth hormone disruption [15,17]. Balance abnormalities, similarly, may occur due to radiation-related injury or disruption to associated structures. In CNS-directed radiation, therapy would be more likely to target balance/stability mediated by cerebellar areas, vestibular pathways, and the brainstem. Beyond radiation, diagnosis-related surgical interventions and history of related concerns (i.e., hydrocephalus) may also be contributory [20]. The reported “medical specialists seen” also reflected differences by site of disease. Such information can help us target specific questions in the future to patients to ascertain key information without burdening the patient or parent proxy reporter.

### 4.5. Social, Educational, and Employment Impact

The majority, 61%, of our patients who were still in school required some form of school support. This is indicative of the wide spectrum of effects the tumor and its treatments have on our childhood cancer survivors. These findings support the establishment of comprehensive systems of aid for the success and well-being of childhood cancer survivors in educational settings. Unfortunately, once a cancer survivor graduates from the school environment, supportive and enabling services are not as readily available, which has implications for employability. The full/part-time employment rate of our responding patients above age 18 (and not school-enrolled) was 80.5%, which is higher than recent average reported employment rates in the U.S. adult survivors of the pediatric cancer population (74%) [21,22]. This may indicate that we are improving the late-effect profile of our pediatric cancer survivors and that the less harmful effects of proton therapy may play a role in that.

### 4.6. Free Expression

The final free-text box responses demonstrated the wide range of interests and accomplishments of the survivor cohort reported for the past year. Though previously established studies have shown the marriage and employment rates of survivors of childhood malignancies to be lower than those of the general population, a large number of patients reported milestones such as engagement/marriage, starting a family, high-level representation in athletic competitions, finding their dream job, or gaining admission into their goal educational program [23,24,25,26,27,28,29]. Though the content of these responses was not quantified for further categorization, the overall information reported by patients show we are making strides in improving our oncologic therapies, and this is consistent with the recent publication of improved quality of life for survivors of pediatric malignancies [29,30,31,32,33,34].

## 5. Conclusions

The Health Status Survey is a short method of data collection that is easily replicable in many environments and has the potential to provide vital information about patient health and social outcomes after treatment. This data can be used to guide practices for survivorship care in healthcare and additional support services in schools. Difficulty of follow up extends beyond radiation therapy to quaternary care centers for multiple specialties, and even beyond this to include vulnerable and/or rural populations who may not be able to consistently attend in-person follow up medical encounters at specialty treatment centers [5]. Future iterations of the survey distributed at quaternary care institutions may consider incorporating emerging machine learning capabilities to aid in predicting which survivors are at higher risk for loss to follow up. This could help institutions increase proactive outreach and improve resource prioritization. Additionally, to reduce manual workload of the institutional team, automations in contact detail updates and integration of HSS responses with electronic health records could strengthen longitudinal data capture. The HSS has the potential to expand into a wide range of patient populations with adapted response options better suited to the treatment and diagnosis of the patient population.

This article is a revised and expanded version of an abstract titled Implementation and Evaluation of a Patient-Reported Health Status Survey for Survivors of Childhood Malignancies Treated with Radiation, which was presented at the 66th Annual Meeting of the American Society for Radiation Oncology (ASTRO) in Washington, D.C., on 30 September 2024 [35]. 

## Figures and Tables

**Table 1:** Patient demographics and tumor subtype breakdowns for CNS and non-CNS radiation-treated cohorts (1a: Demographics, 1b: Tumor subtypes and CSI exposure). (**a**) Patient demographics including age and primary tumor group (CNS vs. non-CNS); (**b**) tumor subtypes for CNS and non-CNS cohorts. Diagnoses occurring in fewer than 5 patients are grouped under “Unspecified/Other.” CNS diagnoses likely to receive craniospinal irradiation (CSI) are noted.

(a)	
**Respondent Demographics**	***n* = 322**
Age at RT (years) ^1^	9.9 (0.88–24.72)
Age at time of survey completion (years) ^1^	18.74 (2.34–35.4)
**Age Status (minor/adult at RT and Survey) ^2^**	
Minor at RT, Minor at Survey	147 (45.7%)
Minor at RT, Adult at Survey	137 (42.5%)
Adult at RT, Adult at Survey	38 (10.2%)
**Tumor Site ^2^**	
Central Nervous System (CNS)	207 (64.3%)
Non-CNS	115 (35.7%)
(**b**)	
**Tumor Site ^2^**	
**Central Nervous System (CNS)**	***n* = 207**
Medulloblastoma/PNET/Pineoblastoma	62 (30.0%)
Glial Tumors/Astrocytoma	28 (13.5%)
Vascular Lesions	10 (4.8%)
Ependymoma	31 (15.0%)
Germ Cell	31 (15.0%)
Craniopharyngioma	15 (7.2%)
Other/Unspecified	30 (14.5%)
**CNS Treatment Subtype**	***n* = 207**
Received CSI (Craniospinal Irradiation)	125 (60.4%)
Did not receive CSI	82 (39.6%)
**Non-CNS**	***n* = 115**
Neuroblastoma	12 (10.4%)
Rhabdomyosarcoma	34 (29.6%)
Sarcoma (Bone)	18 (15.7%)
Chordoma	18 (15.7%)
Sarcoma (Soft Tissue)	7 (6.1%)
Hodgkin’s Lymphoma	7 (6.1%)
Other/Unspecified	19 (16.5%)

^1^ Median (minimum–maximum); ^2^ *n* (% of total respondents).

**Table 2:** Reported contact information updates needed in database by age status of respondent.

Contact Information	Changed Any Information	Total
**Age Status (minor/adult at RT and Survey) ^1^**	185 (57.8%)	322
Minor at RT, Minor at Survey	70 (47.6%)	147
Minor at RT, Adult at Survey	89 (65.0%)	137
Adult at RT, Adult at Survey	27 (71.0%)	38

^1^ *n* (% of total respondents).

## Data Availability

Research data are stored in an institutional repository and will be shared on request to the corresponding author.

## Appendix A

Health Status Survey
